# Severe morbidities associated with induced abortions among misoprostol users and non-users in a tertiary public hospital in Ghana

**DOI:** 10.1186/1472-6874-14-90

**Published:** 2014-07-29

**Authors:** Francis JMK Damalie, Edward T Dassah, Emmanuel SK Morhe, Emmanuel K Nakua, Harry K Tagbor, Henry S Opare-Addo

**Affiliations:** 1 Komfo Anokye Teaching Hospital, Kumasi, Ghana; 2 School of Medical Sciences, Kwame Nkrumah University of Science and Technology, Kumasi, Ghana; 3 Department of Obstetrics and Gynaecology, Komfo Anokye Teaching Hospital, P. O. Box KS 1934, Kumasi, Ghana

**Keywords:** Misoprostol, Client characteristics, Induced abortions, Severe morbidity, Ghana

## Abstract

**Background:**

Misoprostol has become a popular over the counter self-administered abortifacient in Ghana. This study aimed to compare the socio-demographic characteristics and clinical complications associated with misoprostol and non-misoprostol induced abortions among patients admitted to a tertiary public health facility in Ghana.

**Methods:**

This was a cross sectional study conducted at the gynaecological ward of Komfo Anokye Teaching Hospital (KATH), over a four-month period using a structured pre-tested questionnaire. Data were analysed using Chi-square, Fisher’s exact and student t-tests. Factors associated with severe morbidity were examined using Poisson regression with robust error variance to estimate crude and adjusted relative risks (RRs) with 95% confidence intervals (CIs). P < 0.05 was considered statistically significant.

**Results:**

Overall, 126 misoprostol users and 126 misoprostol non-users were recruited into the study. About 71% of the clients had self-induced abortions. Misoprostol users were more likely to be younger (p < 0.001), single (p < 0.001), nulliparous (p = 0.001), of higher educational background (p = 0.001), and unemployed (p < 0.001), than misoprostol non-users. Misoprostol users were more likely than non-users to undergo termination of pregnancy because they wanted to continue schooling (p < 0.001) or were not earning regular income to support a family (p = 0.001). Overall, 182 (72.2%) of the women (79.4% misoprostol users vs. 65.1% misoprostol non-users; p = 0.01) suffered severe morbidity. Nulliparous women (adjusted RR, 1.28; 95% CI, 1.08-1.52) and those who had induced abortion after 12 weeks’ gestation (adjusted RR, 1.36; 95% CI, 1.18-1.57) were at increased risks of experiencing severe morbidity. The association between mode of abortion induction and severe morbidity was not statistically significant (p = 0.06).

**Conclusion:**

Self-induced abortions using misoprostol is a common practice among women in this study; nearly three quarters of them suffered severe morbidity. Nonetheless, severe morbidity among misoprostol users and non-users did not differ significantly but was directly related to the gestational age at which the induced abortions occurred. Health education on the dangers of self-induced abortions and appropriate use of medication abortion could help reduce complications associated with induced abortions in Ghana.

## Background

Each year an estimated 20% (~42 million) of all pregnancies worldwide end in induced abortions, 20 million of which are performed under unsafe conditions [1,2]. Thirteen per cent of maternal deaths worldwide are attributable to unsafe abortions, most of them in developing countries [1,3]. Additionally, an estimated five million women in developing countries are hospitalized each year with complications of induced abortions [4]. Unsafe abortions are frequently carried out by individuals lacking the necessary skill and sometimes self-induced. Africa has one of the highest induced-abortion rates in the world (29 per 1000 women of reproductive age) and more than 90% of these are unsafe [1,2]. These induced abortion rates tend to be higher in settings with high unmet need for contraception and are likely to keep rising unless women’s access to effective contraception and safe abortion care are improved [1].

The abortion law in Ghana was amended in 1985 to make it more liberal. The current law permits abortion carried out by a registered medical practitioner in a registered public or private health facility under the following circumstances: rape, defilement and incest; where the pregnancy poses substantial risk to the physical or mental health of the woman; or in cases of severe fetal anomaly [5,6]. In 2003, a policy to provide abortion care to the full extent of the law was developed. And since 2006, service delivery standards and protocols on prevention and management of unsafe abortion including provision of comprehensive abortion care have been implemented nationwide [7]. Despite the less-restrictive legal environment, about 15 induced abortions per 1000 women of reproductive age are performed in the country each year [8] and two-thirds of these are unsafe [5,9].

In Ghana, induced abortion complications account for 11% of maternal mortality nationwide [8], and up to 12% of gynaecological admissions in leading public tertiary hospitals [10,11]. These voluntary terminations of pregnancy are more common in urban areas and among younger women [5,8,9]. Different methods are used for these terminations. Recent evidence from one study, indicated that over 80% of the women self-induced abortions with misoprostol [11]. Misoprostol is a prostaglandin E_1_ analogue that is licensed for the treatment of peptic ulcer disease. As a prostaglandin, it also causes uterine contractions and cervical softening [12,13]. Misoprostol is employed for labour and abortion induction based on these effects. The declining complication and mortality rates associated with induced abortions in some developing countries especially in Latin America have largely been attributed to the widespread use of misoprostol [3,12-15] and improved health care system [3].

With increasing popularity of misoprostol as an over the counter self-administered abortifacient in Ghana [11], it is imperative that the morbidities associated with its use are investigated. While most studies on induced abortions have compared complications of spontaneous and induced abortions, there is paucity of research on morbidities associated with misoprostol-induced abortions. This study aimed at comparing socio-demographic characteristics and clinical complications associated with misoprostol and non-misoprostol induced abortions among patients admitted to Komfo Anokye Teaching Hospital (KATH), Kumasi, Ghana.

## Methods

This was a cross sectional study conducted at the gynaecological ward of KATH, a 1000-bed capacity hospital and the second largest in the country. The hospital is the main tertiary and referral health facility in the northern sector of the country, serving seven out of the 10 regions of the country. The Obstetrics and Gynaecology Department of the hospital admits about 4,000 gynaecological cases each year.

The department has a comprehensive abortion care unit which provides emergency services such as resuscitation, evacuation of the uterus, administration of oxytocics, antibiotics, and analgesics. All clients are given comprehensive post-abortion care including contraceptive counselling and offer of methods of their choice before they are discharged from the hospital. For all women with abortion complications, the attending physicians explored and routinely documented the clinical history and findings in the client clinical record. These included among others the estimated gestational age, how the index miscarriage started, the treatments sought and medications taken before arrival in the hospital. Methods used to initiate or induce abortion including the use of misoprostol (‘cytotec’, the common name for misoprostol in Ghana) were documented.

Additionally, during the peri-evacuation counselling, a dedicated abortion care counsellor explored and documented the reproductive history of the woman. This included how the index miscarriage occurred, fertility desires of the woman and post-abortion contraception choices. Thus the physician who provided emergency clinical care of the woman, and the dedicated reproductive health counsellor are two main independent sources of information on the type of miscarriage and the methods of induced abortion employed.

All women who were admitted to the gynaecological ward of KATH between June 15 and October 15, 2010 with a history or evidence of induced abortion were eligible for inclusion into the study; women with spontaneous abortions were excluded. Each morning, the medical records of all eligible women who had been treated and were ready for discharge were reviewed by the research team and gynaecological team responsible for the clients to determine the method(s) used by each client to induce abortion. Eligible clients were then categorized into those who had used misoprostol and those who had not used misoprostol to induce abortion in the index pregnancy. Every eligible client who admitted to using any other method apart from misoprostol to induce abortion in the index pregnancy and the next consecutive client who admitted to inducing abortion with misoprostol were selected to participate in the study. Selected clients were approached individually by a trained research assistant (not involved in the care of the client), who explained the purposes of the study to the client and sought her consent to participate in the study. All consenting clients underwent a confidential interview in Twi (vernacular) or English using a pretested structured questionnaire (Additional file 1). Where an eligible woman declined consent/participation, the next eligible and consenting client was recruited. The questions in the questionnaire were not validated but were developed based on the review of relevant current literature on induced abortions.

Pre-testing of the questionnaires was done by administering draft copies to ten patients admitted with abortion related complications at the University Hospital of the Kwame Nkrumah University of Science and Technology (KNUST) also located in the Kumasi metropolis. Necessary corrections and modifications were made to obtain the final questionnaire that was used in the data capture during the survey.

Data were collected on client socio-demographic characteristics, gynaecological and obstetric histories, whether or not the miscarriage was induced, and if so, persons initiating abortion and method(s) of abortion induction used. These questions were open ended to allow respondents state various abortion induction methods employed. Other information gathered were estimated gestational age at the time of abortion and first point of call. The morbidities and treatment associated with the current admission were extracted from the clinical records. The validity of the information given during the interview was crosschecked with those documented in the medical records.

Severe morbidity, the main outcome measure, was defined as the presence in a client of one or more of the following; length of hospital stay more than 24 hours, need for blood transfusion, parenteral antibiotics, laparotomy, pyrexia (temperature ≥ 38°C), peritonitis, shock (blood pressure at time of admission less than 90/60 mmHg) and deranged renal or liver function tests [16-18]. All the variables for severe morbidity were categorized as being severe or not (i.e. binary outcome).

Using Epi Info version 3.5.1 (Centers for Disease Control and Prevention, Atlanta, USA), an estimated sample size of 220 (110 in each group) had over 80% power to detect a relative risk of 1.5 at 95% confidence level, assuming the ratio of misoprostol users to non-users is unity and the prevalence of severe morbidity among non-users of misoprostol is about 40% [17].

Data were double entered into database in Epi Info, cleaned and transferred to Stata version 11.2 (Stata Corp, College Station, Texas, USA) for statistical analyses. Categorical variables were compared using the Chi-square (*χ*^2^) or Fisher’s exact tests as appropriate while continuous variables were compared using student t-tests. Factors associated with severe morbidity were examined using Poisson regression with robust error variance to estimate crude and adjusted relative risks (RRs) with 95% confidence intervals (CIs). This regression technique allows for an unbiased estimate of the RR when the outcome of interest occurs more than 10% of the time [19], as was the case for severe morbidity in this analysis. P < 0.05 was considered statistically significant. All missing values were excluded from the analyses.

Written permissions from the department of Obstetrics and Gynaecology, KATH, to interview patients and access their records were duly obtained. Indeed, these were pre-requisites for ethical clearance Ref: CHRPE/82/09 which was obtained prior to commencement of the study on September 4, 2009. The ethical approval was given by the Committee on Human Research Publications and Ethics of the Kwame Nkrumah University of Science and Technology, Kumasi, Ghana.

## Results and discussion

A total of 344 clients with induced abortion were admitted to the gynaecological ward within the four-month study period. Of these, 199 (57.9%) clients admitted to having used misoprostol to induce their abortion. Overall, 126 misoprostol users and 126 misoprostol non-users who satisfied the inclusion criteria were recruited into the study. Seventy-three (i.e. 199–126) misoprostol users were not recruited into the study; 72 of them were excluded by the sampling strategy and the remaining one declined consent. Also, 19 misoprostol non-users admitted during the study period were discharged and left the ward before they could be approached by the research team.

Table 1 compares the background characteristics of misoprostol users and non-users. Misoprostol users were more likely to be teenagers (p < 0.001), single (89.7% vs. 57.1%; p < 0.001), Christians (94.4% vs. 77.4%; p < 0001), nulliparous (61.9 vs. 34.9%; p = 0.001), and to have completed at least basic (middle or junior high school) level of education (81.5% vs. 61.9%; p = 0.001). They were less likely to be in income-earning employments (27.9% vs. 50.9%; p < 0.001). However, the number of lifetime pregnancies was similar for misoprostol users and non-users (p = 0.16).

**Table 1:** Comparison of background characteristics of misoprostol users and non-users

**Variable**	**Misoprostol users**	**Misoprostol non-users**	**p value**
	**N = 126**	**N = 126**	
	n (%)*	n (%)*	
Age (years)			< 0.001
<20	54 (42.9)	14 (11.1)	
20+	72 (57.4)	112 (88.9)	
Mean (SD)	21.7 (4.9)	25.9 (6.8)	< 0.001
Marital status			< 0.001
Single	113 (89.7)	72 (57.1)	
Married	13 (10.3)	54 (42.9)	
Educational level completed†			0.001
Less than basic	23 (18.6)	48 (38.1)	
Basic education or higher	101 (81.5)	78 (61.9)	
Religion§			< 0.001
Christian	119 (94.4)	96 (77.4)	
Muslim	7 (5.6)	28 (22.6)	
Occupation‡			< 0.001
Income earners	34 (27.9)	60 (50.9)	
Non-income earners	88 (72.1)	58 (49.2)	
Gravidity			0.16
Primigravid	40 (31.8)	30 (23.8)	
Multigravid	86 (68.3)	96 (76.2)	
Parity			0.001
Nulliparous	78 (61.9)	44 (34.9)	
Parous	48 (38.1)	82 (65.1)	

SD, standard deviation.

*Values are given as number (percentage) unless otherwise indicated.

†Basic education: Middle/Junior High School; 2 missing values in the misoprostol users group.

§2 missing values in the misoprostol non-users group.

‡12 missing values (4 in misoprostol users and 8 in misoprostol non-users group).

The reasons for terminating pregnancy varied between users and non-users of misoprostol (Table 2). Misoprostol users were more likely than non-users to undergo termination of pregnancy because they were in school or wanted to continue schooling (p < 0.001) or they were not earning regular income to support a family (p = 0.001). On the other hand, significantly more non-users of misoprostol than users cited no apparent reason for undergoing termination of pregnancy (p < 0.001) or did so because of their desire to limit family size (p = 0.02). No statistically significant differences were observed in terms of relationship problems with male partners among users and non-users of misoprostol.

**Table 2:** Reasons for termination of pregnancy among misoprostol users and non-users

**Reason for abortion**	**Misoprostol users**	**Misoprostol non-users**	**p-value**
	**N = 126**	**N = 126**	
	**n (%)**	**n (%)**	
Wants to continue/still schooling	36 (28.6)	10 (7.9)	<0.001
No regular source of income to support a family	26 (20.6)	8 (6.3)	0.001
Not in a stable relationship	34 (27.0)	35 (27.8)	0.90
Desire to limit family size	23 (18.3)	39 (31.0)	0.02
Medical reasons	4 (3.2)	3 (2.4)	0.90
Accidental medication	-	4 (3.2)	-
No reason stated	3 (2.4)	27 (21.4)	<0.001

Over two-thirds (70.6%) of clients reported that they had self-induced abortions. About 1 in 10 abortions (10.7%) were initiated by health workers (doctors or pharmacists/dispensing technicians). While about 6.4% of clients reported that their abortions were initiated by their male partners, 11.1% admitted that other relations such as relatives, friends, school mates, and co-workers, were the initiators. In 1.2% of the clients, the abortions were initiated by traditional healers. More than two-thirds (68.3%) of non-users of misoprostol used herbal preparations, alcoholic beverages (such as Guinness Foreign Extra Stout), sugar concoctions and some locally prepared blood tonics as abortifacients. The identifiable herbal preparations were commonly locally prepared concoctions of leaves or barks of some plants in alcohol. About 1 in 5 misoprostol non-users had instrumentation, mainly dilation and curettage or manual vacuum aspiration. The remaining 11.1% of misoprostol non-users took over-the-counter drugs (ergometrine or norethisterone enanthate tablets) to induce abortion.

Overall, 182 (72.2%) clients had severe morbidities [79.4% misoprostol users vs. 65.1% non-users; (p = 0.01)] (Table 3). However, comparing the individual categories of severe morbidities, the differences between misoprostol users and non-users were not statistically significant (all p-values > 0.05). The duration of admission ranged from less than a day to 24 days (median duration 2 days; interquartile range 1–4 days).

**Table 3:** Comparison of severe morbidities between misoprostol users and non-users

**Morbidity**	**Misoprostol users**	**Misoprostol non-users**	**p value**
	**N = 126**	**N = 126**	
	**n (%)**	**n (%)**	
Severe hemorrhage	46 (36.5)	34 (27.0)	0.10
Septicaemia	53 (42.1)	40 (31.8)	0.09
Deranged liver or kidney function tests	10 (7.9)	4 (3.2)	0.17*
Laparotomy	1 (0.79)	2 (1.6)	1.00*
Prolonged hospital stay	74 (58.7)	60 (47.6)	0.08
Any severe morbidity	100 (79.4)	82 (65.1)	0.01

*Fisher’s exact test.

On univariable analysis, (Table 4), misoprostol users were more likely to suffer severe morbidities compared to non-users (unadjusted RR, 1.22; 95% CI, 1.04-1.43; p = 0.01), as were teenagers compared to older women (unadjusted RR, 1.17; 95% CI, 1.01-1.36; p = 0.04), single compared to married women (unadjusted RR, 1.42; 95% CI, 1.13-1.78; p = 0.003), and nulliparous compared to parous women (unadjusted RR, 1.27; 95% CI, 1.09-1.48; p = 0.002). The strongest predictor of severe morbidity was the gestational age at which termination occurred (p < 0.001).

**Table 4:** Univariable analysis of association between background characteristics and severe morbidities

**Variable**	**Severe morbidity**	**Unadjusted RR**	**p value**
	**n (%)**	**(95% CI)**	
Cohort			0.01
Misoprostol non-user	82 (65.1)	1	
Misoprostol user	100 (79.4)	1.22 (1.04, 1.43)	
Age (years)			0.04
<20	55 (80.9)	1.17 (1.01, 1.36)	
20+	127 (69.0)	1	
Marital status			0.003
Single	145 (78.4)	1.42 (1.13, 1.78)	
Married	37 (55.2)	1	
Educational level completed			0.52
Less than basic	49 (69.0)	1.06 (0.89, 1.23)	
Basic education or higher	131 (73.2)	1	
Religion			0.26
Christian	151 (71.6)	1	
Muslim	31 (79.5)	1.11 (0.93, 1.33)	
Occupation			
Income earners	62 (66.0)	1	0.13
Non-income earners	110 (75.3)	1.14 (0.96, 1.36)	
Gravidity			0.89
Primigravid	51 (72.9)	1.01 (0.85, 1.20)	
Multigravid	131 (72.0)	1	
Parity			0.002
Nulliparous	99 (81.2)	1.27 (1.09, 1.48)	
Parous	83 (63.8)	1	
Estimated gestational age at presentation			<0.001; p trend = 0.06
<9 weeks	123 (71.9)	1	
9-12 weeks	31 (63.3)	0.88 (0.70, 1.11)	
>12 weeks	27 (93.1)	1.29 (1.13, 1.48)	

RR, Relative risk; CI, Confidence interval.

On multivariable analysis (Table 5), the estimated gestational age at presentation (p < 0.001) and parity (adjusted RR, 1.28; 95% CI, 1.08-1.52; p = 0.004) were independent risk factors for severe morbidity. Nulliparous women were 1.3 times more likely than parous women to suffer severe post-abortion morbidity. Women who terminated their pregnancies after 12 weeks’ gestation were about 1.4 times more likely to suffer severe morbidity than their counterparts who induced abortions before nine weeks of gestation (adjusted RR: 1.36; 95% CI: 1.18-1.57). There was a significant linear trend towards suffering severe morbidity with increasing gestational age (p trend = 0.02) at which induced abortion occurred. There was no significant association between misoprostol use and severe morbidity among the women studied (p = 0.06).

**Table 5:** Final multivariable model for factors associated with severe morbidities

**Variable**	**Severe morbidity**	**Adjusted RR**^**a**^	**p value**
	**n (%)**	**(95% CI)**	
Cohort			
Misoprostol non-user	82 (65.1)	1	0.06
Misoprostol user	100 (79.4)	1.17 (0.99, 1.38)	
Age (years)			0.88
<20	55 (80.9)	0.99 (0.83, 1.17)	
20+	127 (69.0)	1	
Parity			0.004
Nulliparous	99 (81.2)	1.28 (1.08, 1.52)	
Parous	83 (63.8)	1	
Estimated gestational age at presentation			<0.001; p trend =0.02
<9 weeks	123 (71.9)	1	
9-12 weeks	31 (63.3)	0.88 (0.70, 1.11)	
>12 weeks	27 (93.1)	1.36 (1.18, 1.57)	

RR, Relative risk; CI, Confidence interval.

^a^Adjusted for all variables in the table.

The study compared the demographic and clinical characteristics of misoprostol users and non-users among clients admitted with induced abortion complications at the second leading tertiary hospital in Ghana. Consistent with characteristics of induced-abortion clients in Ghana [8,9,11,20,21] and other countries [15,22], we found that misoprostol users were younger, not married, nulliparous, and had better educational background than non-users. Gestational age at which pregnancy was terminated and being nulliparous were independent risk factors for severe morbidity following termination of pregnancy. The association between misoprostol use and severe morbidity was not statistically significant.

About 60% of abortions in this study were induced with misoprostol, confirming earlier reports that misoprostol had become a popular and relatively cheap over the counter abortifacient among women in Kumasi [11], and possibly in Ghana. The figure is however, lower than 83% reported earlier in the same centre [11], but much higher than the 5-16% reported in the 2007 Ghana Maternal Health Survey [8]. A number of reasons may account for the higher proportion of misoprostol-induced abortions in the study centre. Although the law on abortion in Ghana is quite liberal and permits abortion under certain circumstances, most physicians in public hospitals and especially at the study centre are unwilling to initiate termination of pregnancies [8,23]. However, as a tertiary centre clients who experience complications after seeking abortion care elsewhere are likely to be referred for post-abortion care.

About 90% of induced-abortions in this study were initiated by the clients themselves or unqualified or untrained persons. Such clients reported to this tertiary facility when they encountered problems or complications [12,13,17,24]. Moreover, most terminations of pregnancies performed by qualified medical professionals under safe conditions are less likely to suffer complications [21] and will not be referred/report to KATH. The difference in reported prevalence of self-induced abortion could also be due to more under-reporting of clandestine abortion procedures than abortions procured from medical professionals [21]. Compared to the earlier study by Konney *et al*. [11], both the number of clients with induced abortions and the proportion of misoprostol-induced abortions are lower in this study. As observed in Brazil [12,15], it is conceivable that women in the study area might have gained experience with the drug over time resulting in usage of safer and more effective doses and consequently fewer complications and admissions arising from self-induced abortions with misoprostol.

More women who wanted to continue schooling or were not earning income regularly resorted to using misoprostol to induce abortion. To these non-income earners misoprostol may be cheaper than other methods of procuring induced abortion. The high prevalence of self-induced abortion could be due to high level of social stigma associated with abortion care in Ghana [5,9,21,23]. It could also be motivated by the fact that, by starting the miscarriage the client can have access to health facilities that do not offer elective abortion care. Additionally, the clients with abortion complications can obtain emergency care under the free maternity care programme of the National Health Insurance Scheme (NHIS) which covers abortion related complications but not elective termination of pregnancy. Thus, once misoprostol can be acquired without prescription, women can procure and self-administer it to terminate pregnancies without professional guidance or supervision [12,13,15,24].

On the other hand, for the non-users of misoprostol, induced-abortions were mainly considered because they did not want any more children or had no obvious reasons for the termination. These reasons are consistent with findings from other studies on induced abortions in Ghana [8,9,20,21]. While we did not investigate contraceptive use in this study, about 35% of married women in Ghana have an unmet need for contraception [8].

Nearly three-quarters of women in this study had severe morbidities. This is much higher than the 34% severe complication rate reported by Rees et al. in South Africa [16]. Though similar to the complication rate reported in Cambodia [17], the rate is still unacceptably high in a country with a non-restrictive abortion law. Non availability of and poor access to safe abortion services in most communities may contribute to the high rate of severe morbidity in this study. For, even in the context of a liberal legal regime, if accessibility to safe abortion is limited, it would remain the case that abortion-related morbidity would persist [5,6]. Almost all the women had attempted termination of pregnancy either by themselves or unqualified persons prior to reporting to this tertiary facility for the treatment of their abortion-related complications. Notwithstanding the implementation of the policy on comprehensive abortion care, unsafe abortions continue to flourish due to a number of reasons. These may include slow translation of the law into policy, service protocols and standards, limited understanding of the law and its implications by many communities and providers, ignorance of most Ghanaian women about their legal rights to abortion and the reluctance of most providers to offer elective abortion services. For many women, safe abortion care is quite expensive as the service is mainly offered by few private practitioners who charge ridiculously high fees. Thus, cheaper alternatives of inducing abortion such as the self – administration of misoprostol are easily adopted by the women with unwanted pregnancy [5,6,11,23]. Accessibility to safe abortion services is further hampered by the fact that only treatment for abortion related complications, but not elective abortions, is covered by the NHIS.

Generally, risk of complications associated with all forms of abortion increases with gestational age, rising exponentially after the first trimester, even when terminations are performed under the best of circumstances [25,26]. The risk is even higher when the abortions are performed by individuals lacking the necessary skill or in an environment that does not meet the minimum medical standards. Although terminations after the first trimester constitute the minority of abortion cases, they account for the majority of serious abortion-related complications including death, particularly in settings where unsafe abortions are common [25-27]. The finding suggests that women who want to end unwanted pregnancies should initiate the process early in the pregnancy to avoid experiencing severe morbidities.

In agreement with previous studies [28,29], we found that women who had not given birth previously were at increased risk of severe morbidity compared to their parous counterparts. This is possibly because more nulliparous women tend to retain more products of conception compared to parous women, thereby increasing their risk of severe morbidity [28].

Whereas some studies have reported reduced risks of severe complication with misoprostol use compared to other methods of inducing abortion [12,13,15], we did not observe such an association. It is worth noting that success or complications associated with using misoprostol to abort pregnancies depend on a number of inter-related factors such as whether misoprostol is used as a single agent or not, the dose, schedule, and route(s) of administration as well as the gestational age at abortion (including an accurate assessment of the same) [12,13,15]. The effects of most of these additional factors were not examined by this study.

The study has some limitations. Results from this urban tertiary referral facility are not representative of the general population. It is likely that women’s experience with and access to care for induced abortion and its complications in rural settings will be different. Both the estimates for induced abortions and the proportion of misoprostol induced abortions are likely to be higher than what was reported in the study due to misclassification bias. In settings where induced abortion is stigmatized, women - especially those with complications - are likely to report such incidents as spontaneous rather than induced. However, this is likely to be minimal in this study as questions on induced abortion were posed by experienced physicians and counsellors involved in abortion care. In addition, questions on the dose and routes of administration of misoprostol, concurrent use of other agents as well as contraceptive use were not included in our study.

Further research is required to explore women’s motivation for using misoprostol as an abortifacient as well as their experiences including the cost, accessibility, safety, dosing regimens and the routes of administration. There is also need to assess the long-term impact of the use of misoprostol on complications related to induced abortions.

## Conclusion

Self-induced abortions using misoprostol is a common practice among women in this study. Misoprostol users were more likely to be young, single, nulliparous, unemployed and of higher educational background than non-users. Nearly three quarters of women with induced abortions suffered severe morbidity. Nulliparous women were at greater risk of suffering severe abortion related morbidity than parous women. Nonetheless, severe morbidity among misoprostol users and non-users did not differ significantly but was directly related to the gestational age at which the induced abortions occurred. Health education on the dangers of self-induced abortions, appropriate use of medication abortion and the provision of accessible and safe comprehensive abortion care at early gestation could help reduce complications associated with induced abortions in Ghana.

## Abbreviations

KATH: Komfo Anokye Teaching Hospital; RR: Relative risk; CI: Confidence interval; NHIS: National Health Insurance Scheeme.

## Competing interests

The authors declare that they have no competing interests.

## Authors’ contributions

FJMKD, ETD, ESKM, EKN, HKT and HSOA conceived and designed the study. EKN and ETD analyzed the data. FJMKD, ETD and ESKM wrote the first draft of the manuscript. All authors read and approved the final manuscript.

## Pre-publication history

The pre-publication history for this paper can be accessed here:

http://www.biomedcentral.com/1472-6874/14/90/prepub

## Supplementary Material

Additional file 1 Comparison of abortion morbidity between misoprostol users and non-users in Kumasi, Ghana. Click here for file
