# Supplementary material for: Severe morbidities associated with induced abortions among misoprostol users and non-users in a tertiary public hospital in Ghana
Source: BMC Womens Health. 2014 Jul 29;14:90. doi: 10.1186/1472-6874-14-90 (PMC4118200; doi:10.1186/1472-6874-14-90)
Supplement: Additional file 1 — Comparison of abortion morbidity between misoprostol users and non-users in Kumasi, Ghana. [file 1472-6874-14-90-S1.docx]

MS **2208805121178062 - Additional file 1**

COMPARISON OF ABORTION MORBIDITY BETWEEN MISOPROSTOL USERS AND NON-USERS IN KUMASI, GHANA

QUESTIONNAIRE

A. SOCIO-DEMOGRAPHY

Serial No________________

1. Age _________________
2. Gravidity: gravida_______para_______ + _______ (spontaneous/induced/missed abortions and ectopic pregnancies)

Occupation (***please tick one***)

1. Unemployed vii. Semi skilled (hair dresser,
2. Housewife seamstress, sales girl)
3. Farmer
4. Artisan apprentice viii. Professional (banker, teacher,
5. Student administrator)
6. Trader/businesswoman ix. Others
7. Religion ( ***please tick one)***
8. Christian iii. Traditional religion
9. Muslim iv. Others (specify) ____________
10. Marital Status: ***(please tick one)***
11. Single
12. Married/in a relationship
13. Educational status (***please tick one***)
14. No-formal iv. Senior High School
15. Primary v. Tertiary
16. Middle/Junior High School

B. PRESENTATION

First point of call

i. KATH

ii. A maternity home or health centre

iii. Other hospitals

**C. METHODS USED FOR ABORTION INDUCTION AND REASONS**

**1. Methods used to initiate abortion**

**Please specify the procedure or medication used to initiate the abortion**

NB: Please , probe further to find out the exact procedure or medication used e.g. if patient stated that she was taken to theatre, determine whether Dilatation and Curettage (D&C) or Manual Vacuum Aspiration (MVA) was used and if a patient mentioned medications, determine the exact medication used. Similarly specify the name of herbal medications used.

2. Who initiated the abortion?

1. Self v. Nurse/midwife
2. Partner vi. Medical assistant
3. Traditional healer/herbalist vii. Doctor
4. Pharmacist/dispensing technician viii. Others (specify)--------------

3. Reasons for termination of pregnancy

1. Wish to continue/still schooling vi. Contraceptive failure
2. Not married vii. Medical reasons
3. Not in a stable relationship viii. Accidental medication
4. Unemployed ix. No reason stated
5. To limit family size x. Others (specify)

**RECORDS REVIEW GUIDE**

D. Gestation at which abortion was induced (by USG, LMP, uterine size estimation)

State in weeks____________________________

E. ABORTION RELATED COMPLICATIONS

1. i. Temperature at time of admission _____________________ ̊C

ii. Pulse at time of admission ___________________________ beats per minute

iii. Blood pressure at time of admission __________________ mmHg

2. Main complications associated with the abortion (***tick as many as may apply)***

SYMPTOMS/SIGNS YES NO

1. Severe abdominal tenderness/peritonitis
2. Shock
3. Offensive products of conceptus evacuated
4. Uterine perforations
5. Bladder or bowel injuries
6. Cervical or vaginal laceration
7. Lab evidence of impaired kidney or liver function

3. Definitive treatment/medications given (***tick as many as may apply )***

Treatment Yes No

1. i. Blood transfussion
2. ii. Parenteral antibiotics
3. iii. Laparotomy
4. iv. Uterine repair
5. v. Bowel repair
6. vi. Hysterectomy

4. Duration of hospitalization.

i. Less than 24hrs ii. 24hrs or more
